# Editorial: Regulated cell death pathways in the initiation and reversal of inflammation and tissue repair

**DOI:** 10.3389/fimmu.2024.1367215

**Published:** 2024-01-22

**Authors:** Hsin-Hou Chang

**Affiliations:** Department of Molecular Biology and Human Genetics, Tzu-Chi University, Hualien, Taiwan

**Keywords:** regulated cell death, inflammation and tissue repair, drug target, apoptosis, pyroptosis, necroptosis, ferroptosis, autophagy

The elucidation of regulated cell death (RCD) pathways has emerged as a cutting-edge area of investigation with profound implications for understanding the intricate balance between inflammation and tissue repair. These diverse cell death mechanisms are now recognized as interconnected regulators of immune responses and tissue homeostasis. The current scientific discourse strives to unravel the intricate signaling networks governing the initiation and resolution of inflammation through these RCD pathways, offering promising avenues for therapeutic interventions aimed at manipulating immune responses and optimizing tissue healing in various pathological contexts. A deeper understanding of these RCD processes will require new strategies and methodologies, which will take us to a new era of therapeutics. Considering the rapid growth of interest in the RCD research, we are delighted to present a focused issue featuring 7 specifically commissioned cutting-edge research and review articles that shed light on recent developments in the initiation and reversal of inflammation and tissue repair.

## Cutting-edge experimental models exploring inflammation linked with regulated cell death

Having practical experimental models is essential for studying mechanisms and developing drugs. Son et al. delve into the intricate relationship between particulate matter 10 (PM10) exposure and inflamed intestines, emphasizing a potential exacerbation of inflammatory disorders. Utilizing two-dimensional (2D) intestinal epithelial cell culture and 3D organoid models, the study meticulously examines PM10-induced alterations, revealing pathological features like inflammation, decreased intestinal markers, and compromised barrier function. Notably, PM10 exposure leads to a severe disturbance in peptide uptake, attributed to interference with calcium signaling, protein digestion, and absorption pathways. The findings underscore the significance of 2D and 3D models as robust platforms for elucidating the causal link between PM exposure and abnormal human intestinal functions, offering valuable insights for future research and potential interventions.

Carignon et al. investigated the role of alveolar epithelial type 1 (AT1) cells in inflammation and interstitial fibrosis in chronic lung diseases. Employing an inducible model of AT1 cell depletion, the study demonstrates the impact of repeated injury, revealing inflammation, increased tissue repair markers, and interstitial pulmonary fibrosis. This approach sheds light on the role of AT1 cells in lung inflammatory diseases, providing a foundation for identifying therapeutic targets. The study represents a crucial step forward in understanding the multifaceted pathogenesis of chronic lung diseases, offering potential avenues for targeted interventions and improved patient outcomes.

Terren et al. addressed the complexities of cancer immunotherapy, this research focuses on cytokine-induced memory-like (CIML) natural killer (NK) cells and their mitochondrial dynamics. Stimulation with interleukin (IL)-12/15/18 results in decreased NK cell viability, altered mitochondrial morphology, and increased superoxide levels. Despite slight mitophagy impairment, heightened autophagic flux is observed, providing insights into potential strategies for enhancing the therapeutic efficacy of IL-12/15/18-stimulated NK cells. The study contributes valuable knowledge to CIML NK cell biology, paving the way for future advancements in cancer immunotherapy by addressing mitochondrial fitness and cell viability.

In the realm of heart transplantation, Guo et al. unravels the intricate networks of lncRNA-miRNA-mRNA contributing to T cell-mediated acute rejection (AR). Leveraging a mouse heart transplantation model, the research identifies differentially expressed RNAs, unveiling specific pathways involved in cardiac functions and immune responses. The proposed ceRNA networks, validated through various pathways, offer potential diagnostic gene biomarkers for AR. The study’s comprehensive analysis opens new avenues for understanding and addressing AR after heart transplantation, emphasizing the significance of lncRNA-mediated ceRNA networks as potential biomarkers in this critical clinical context.

## Pathways governing regulated cell death and potential therapeutic implications

It is essential to identify the molecular mechanisms and formulate therapeutic strategies to prevent the inadvertent activation of RCD in the context of diseases. Ke et al. reviewed the intricate relationship between diabetes mellitus and chronic inflammation, focusing on diabetic complications such as ophthalmopathy, cardiovascular issues, and nephropathy. With diabetes-related complications being a significant cause of disability and death, the study delves into the increasing use of anti-inflammatories in combination therapy. It emphasizes the potential of targeting key regulators, specifically receptor-interacting serine/threonine-kinase-1 (RIPK1) and RIPK3, in managing inflammation related to diabetes complications. This review provides a contemporary summary of the mechanism of action and drug development of RIPK1 and RIPK3, shedding light on their pivotal roles in chronic inflammation and immunity. The exploration of selective RIPK1 and RIPK3 inhibitors as anti-inflammatory therapeutic agents for diabetic complications adds a valuable perspective to the ongoing research in this domain.

Gasdermins, a family of pore-forming proteins, take center stage in Slaufova et al.’s comprehensive review, emphasizing their critical roles in inflammatory diseases and cancer. The study elucidates the complex process of gasdermin activation and pore formation, particularly their association with inflammation induced by caspase-1. Focusing on the diverse cellular consequences, the review addresses species, cell type, and membrane-specific variations in gasdermin effects. A special emphasis on keratinocytes in the skin, expressing all members of the gasdermin family, raises intriguing questions about their roles, activation mechanisms, and potential crosstalk. The review provides a current understanding of gasdermins, offering insights into their implications in skin-related immunity and diseases.

Sepsis, a life-threatening condition with high incidence and mortality, is explored in Zhong and Yin’s review, focusing on the crucial role of immunosuppression in its pathophysiology. The study systematically presents the mechanisms of immune dysregulation in sepsis, highlighting the involvement of the programmed cell death 1 (PD-1) signaling pathway in immunosuppression. The review elucidates the expression and regulatory effects of the PD-1 pathway on immune cells associated with sepsis. Current research developments and prospects for applying the PD-1 signaling pathway in immunomodulatory therapy for sepsis are specified, providing a comprehensive overview of the potential therapeutic strategies and guiding future research directions.

With the growing prevalence of inflammatory diseases and the necessity for tissue repair, understanding the regulation of RCD activation in diverse disease contexts is crucial at a molecular level. The insights from this Research Topic could provide valuable perspectives for future research and the development of therapeutic approaches.

## Author contributions

HC: Writing – original draft, Writing – review & editing.

